# Usability and Efficacy of Artificial Intelligence Chatbots (ChatGPT) for Health Sciences Students: Protocol for a Crossover Randomized Controlled Trial

**DOI:** 10.2196/51873

**Published:** 2023-11-24

**Authors:** Mirella Veras, Joseph-Omer Dyer, Morgan Rooney, Paulo Goberlânio Barros Silva, Derek Rutherford, Dahlia Kairy

**Affiliations:** 1 Health Sciences Carleton University Ottawa, ON Canada; 2 Centre for Interdisciplinary Research in Rehabilitation of Greater Montreal Montréal, QC Canada; 3 École de Réadaptation, Faculté de Médecine Université de Montréal Montréal, QC Canada; 4 Groupe Interdisciplinaire de Recherche sur la Cognition et le Raisonnement Professionnel, Faculty of Medicine Université de Montréal Montréal, QC Canada; 5 Teaching and Learning Services Carleton University Ottawa, ON Canada; 6 Centro Universitário Christus Fortaleza, Ceara Brazil; 7 School of Physiotherapy Dalhousie University Halifax, NS Canada; 8 Institut Universitaire sur la Réadaptation en Déficience Physique de Montréal, Centre Intégré Universitaire de Santé et Services Sociaux du Centre-Sud-de-l’Île-de-Montréal Montréal, QC Canada

**Keywords:** artificial intelligence, AI, health sciences, usability, learning outcomes, perceptions, OpenAI, ChatGPT, education, randomized controlled trial, RCT, crossover RCT

## Abstract

**Background:**

The integration of artificial intelligence (AI) into health sciences students’ education holds significant importance. The rapid advancement of AI has opened new horizons in scientific writing and has the potential to reshape human-technology interactions. AI in education may impact critical thinking, leading to unintended consequences that need to be addressed. Understanding the implications of AI adoption in education is essential for ensuring its responsible and effective use, empowering health sciences students to navigate AI-driven technologies’ evolving field with essential knowledge and skills.

**Objective:**

This study aims to provide details on the study protocol and the methods used to investigate the usability and efficacy of ChatGPT, a large language model. The primary focus is on assessing its role as a supplementary learning tool for improving learning processes and outcomes among undergraduate health sciences students, with a specific emphasis on chronic diseases.

**Methods:**

This single-blinded, crossover, randomized, controlled trial is part of a broader mixed methods study, and the primary emphasis of this paper is on the quantitative component of the overall research. A total of 50 students will be recruited for this study. The alternative hypothesis posits that there will be a significant difference in learning outcomes and technology usability between students using ChatGPT (group A) and those using standard web-based tools (group B) to access resources and complete assignments. Participants will be allocated to sequence AB or BA in a 1:1 ratio using computer-generated randomization. Both arms include students’ participation in a writing assignment intervention, with a washout period of 21 days between interventions. The primary outcome is the measure of the technology usability and effectiveness of ChatGPT, whereas the secondary outcome is the measure of students’ perceptions and experiences with ChatGPT as a learning tool. Outcome data will be collected up to 24 hours after the interventions.

**Results:**

This study aims to understand the potential benefits and challenges of incorporating AI as an educational tool, particularly in the context of student learning. The findings are expected to identify critical areas that need attention and help educators develop a deeper understanding of AI’s impact on the educational field. By exploring the differences in the usability and efficacy between ChatGPT and conventional web-based tools, this study seeks to inform educators and students on the responsible integration of AI into academic settings, with a specific focus on health sciences education.

**Conclusions:**

By exploring the usability and efficacy of ChatGPT compared with conventional web-based tools, this study seeks to inform educators and students about the responsible integration of AI into academic settings.

**Trial Registration:**

ClinicalTrails.gov NCT05963802; https://clinicaltrials.gov/study/NCT05963802

**International Registered Report Identifier (IRRID):**

PRR1-10.2196/51873

## Introduction

### Background

The integration of artificial intelligence (AI) into the education field has led to many possibilities, benefiting both educators and students [1]. AI takes on various roles as an intelligent tutor, a collaborative learning partner, a valuable tool, and even an adviser in influencing educational policies [2,3]. The multidimensional role of AI in education, as demonstrated by the potential for dynamic and adaptive learning experiences, aligns with ChatGPT’s capacity to operate as a valuable educational tool. ChatGPT is a generative AI that can generate complex answers to questions asked by the user. It has been described as a tool offering numerous benefits in the field of education [4]. It serves as a platform for enhancing students’ writing skills through features such as text completion, translation, and summarization tools. Educators can integrate ChatGPT into their courses to customize the learning journey for their students by considering their preferred learning methods and existing proficiency levels [4].

An exploratory survey examining the use of ChatGPT in various domains, including education, health care, and research, revealed both positive and negative impacts [5]. On the positive side, ChatGPT was found to level the playing field for students facing language challenges, enhance comprehension by providing additional details, and offer valuable assistance in crafting writing assignments. However, the study also uncovered negative consequences, such as the potential for inaccuracies, concerns about replacing hard work with automated solutions, and the possible discouragement of critical thinking skills. In addition, questions remained regarding academic integrity and students’ willingness to disclose their use of ChatGPT. These findings emphasize the need for careful consideration and guidance when integrating AI technologies such as ChatGPT into various fields [5].

It is crucial to acknowledge that integrating AI into education can result in unintended consequences [6]. The use of ChatGPT, while presenting remarkable potential, has sparked discussions regarding its potential negative implications. Concerns have been raised regarding its potential to promote academic dishonesty, stifle creativity, and diminish critical thinking abilities [7]. Numerous legitimate concerns encompass a spectrum of challenges. These include the potential for generating inaccurate content and ethical dilemmas such as the imminent risk of bias, instances of plagiarism, and complex copyright matters [8]. The findings of a systematic review exploring the application of ChatGPT in health care education, research, and practice revealed that ethical concerns were prevalent in 33 out of 60 records. These concerns, particularly the risk of bias and plagiarism, often revolved around data privacy and security issues [8]. This underscores the significance of investigating students’ perceptions of ethics and accountability concerning the use of ChatGPT in health sciences.

Within the health sciences domain, the successful use of AI relies on several key factors. First, usability is paramount. For example, chatbots and AI programs designed to simulate conversations with human users must be user-friendly, ensuring that students, health care professionals, and patients can interact effortlessly. Second, efficiency is critical because these chatbots should streamline processes, reduce workload, and provide rapid and accurate responses [9]. Finally, the overall effectiveness of these chatbots plays a pivotal role, as they need to deliver tangible benefits such as improved patient care, enhanced diagnostic accuracy, and increased efficiency in health care operations. Consequently, the usability, efficiency, and overall effectiveness of chatbots in health sciences are vital aspects that warrant careful consideration and optimization for their successful integration into the field [8].

In this study, the term *usability* is defined as “a quality attribute that assesses how easy user interfaces are to use.” The word *usability* also refers to “methods for improving ease-of-use during the design process” [10]. The 5 components of usability are learnability (how easy is it for users to accomplish basic tasks the first time they encounter the design?); efficiency (once users have learned the design, how quickly can they perform the tasks?); memorability (when users return to the design after a period of not using it, how easily can they reestablish proficiency?); errors (how many errors do users make, how severe are these, and how easily can they recover from them?); and satisfaction (how pleasant is it to use the design?) [10]. The evaluation of ChatGPT’s usability will be conducted in health sciences students, as assessing the usability of this AI holds significance due to the potential impact on adopting this technology in the training of these students.

Over the past few years, extensive research has investigated the usability of ChatGPT across various domains. A study assessing ChatGPT’s usability in formal English language learning revealed promising outcomes. The findings showed that ChatGPT is an effective tool for formal English language education, excelling in various learning tasks encompassing conversation, writing, grammar, and vocabulary enhancement [11]. A systematic mapping study examining the usability of chatbots and human-computer interaction systems examined the field exhaustively, drawing on numerous references. Of the 170 studies that met the inclusion criteria, 21 were primary studies. The authors’ conclusions highlighted the emerging nature of chatbot usability research, which mainly comprises surveys, informal experimental studies, and usability tests. They emphasized the need for more formal experiments to rigorously gauge the user experience, leveraging these findings to design guidelines that prioritize usability [12]. To our knowledge, no randomized controlled trial (RCT) has assessed the usability and students’ perceptions of ChatGPT use within the health sciences.

### Study Objectives

#### Primary Objectives

We aim to explore the usability and effectiveness of ChatGPT compared with conventional web-based tools as a supplementary assistance tool to enhance undergraduate health sciences students’ learning experiences and outcomes.

#### Secondary Objectives

Our secondary objectives are to (1) explore students’ perceptions and experiences with ChatGPT as a learning tool; (2) explore the potential benefits of or barriers to using ChatGPT to improve students’ writing abilities in the health sciences; and (3) investigate students’ perceptions of accountability, ethical considerations, and equity concerns related to the use of AI in education.

### Study Hypotheses

The study’s hypotheses are as follows:

- *Null hypothesis:* there is no significant difference in technology usability and learning outcomes between students who use ChatGPT (group A) and those who use conventional web-based tools (group B) for accessing resources and completing assignments.
- *Alternative hypothesis:* there is a significant difference in technology usability between students who use ChatGPT (group A) and those who use conventional web-based tools (group B) for accessing resources and completing assignments.
- *Exploratory hypothesis:* the learning outcomes of students who use ChatGPT (group A) will significantly differ from those of students who use conventional web-based tools (group B) for completing their assignments. This hypothesis proposes that the choice of intervention (ChatGPT or conventional web-based tools) may influence the learning outcomes and explore the interaction between usability and learning outcomes.

## Methods

### Study Design

**Figure 1:**
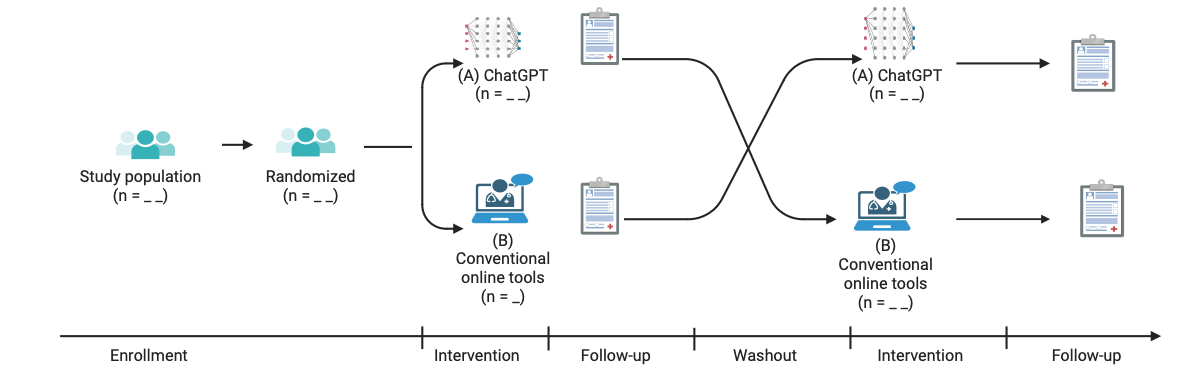
Crossover randomized controlled trial study design on the usability and efficacy of ChatGPT for health sciences students.

We will use a randomized crossover design to ensure equity in the learning experience for all students. This involves assigning students to use ChatGPT (group A) or conventional web-based learning tools (group B) to access resources and assist them in completing their assignments (Figure 1). To address the potential biases associated with the reporting of results using RCT design, we will adhere to 2 specific guidelines: the CONSORT (Consolidated Standards of Reporting Trials) and its extensions. The CONSORT statement was introduced in 1996 to enhance the quality of clinical trial reporting [13]. However, critics observe that the reporting of crossover trials has been inconsistent and incomplete. To address this issue, the CONSORT 2010 statement was extended to provide recommendations for reporting randomized crossover trials, including a specific checklist [14]. This CONSORT extension for crossover trials includes 14 additional items aimed at improving the reporting of such trials. Furthermore, our report will also draw upon the CONSORT-AI, an extension of the CONSORT 2010 statement published in 2020, which focuses on the evaluation of RCTs involving AI [15].

### Setting

This crossover RCT will be conducted on the web and will include health sciences students who are registered in a course on chronic diseases and disability, a third-year undergraduate course at Carleton University in Ottawa, Canada.

Eligible participants will receive comprehensive information about the trial, including the study’s purpose and procedure, through both verbal communication and written materials. To address ethical concerns regarding power dynamics between those responsible for the study and those who may be involved in student teaching and assessment (ie, professors) and students, we opted to involve an individual who is independent of the research team in the recruitment process (ie, a teaching assistant [TA] not involved in other aspects of the study). This decision aims to minimize any potential bias or influence that could arise from direct professor involvement in the recruitment and research. By introducing a TA to be involved in recruitment, we strive to create a more equitable research environment and mitigate any power imbalances that may exist between professors and students. This approach helps ensure that the study is conducted fairly and unbiasedly, fostering a research environment that prioritizes equal opportunities for all participants. Both groups will simultaneously work on an assignment.

### Participants

#### Inclusion Criteria

The inclusion criteria consist of health sciences undergraduate students who are aged ≥18 years and of any gender identity at Carleton University, Canada. They must be registered in a course on chronic diseases and disability during fall of 2023.

Students who responded to the advertisements and consented to participate in the study will be included. Participant recruitment will involve the TA who is not associated with the research project and does not hold any power or authority over the students. The research assistant (RA) will be responsible for overseeing the consent process. They will ensure that informed consent is obtained from the students voluntarily and without undue influence.

#### Exclusion Criteria

Students who do not provide informed consent are not included in the study.

### Recruitment

Recruitment will be conducted in September 2023, followed by interventions and assessments in October and November 2023. All students registered in the course will participate in the learning activities (ie, course assignments) included in this study, as these activities are mandatory for completing the course. They will be asked to give their informed consent for the data generated by their participation in the course assignments to be used for the purposes of the study and for web-based questionnaires to be sent to them to gather information about their learning experience while performing the assignments.

The students will be identified from the class list in Brightspace (Desire2Learn; the digital learning environment of the course), and all students who express their consent will be eligible for inclusion. Upon providing consent, the students will receive an invitation email containing instructions to access and complete the web-based questionnaires through the Qualtrics platform (Qualtrics International Inc). If there is no response within 2 weeks of the initial invitation, a follow-up reminder email will be sent to nonresponding students.

### Sample Size

Recently, Luna et al [16] evaluated the performance of an AI compared with that of a group of physiotherapists in a squat exercise–related quiz and found that the AI demonstrated a sensitivity of 84% in providing correct responses. Assuming a minimum sensitivity of 50% as the null hypothesis, we estimated that 45 participants need to be evaluated to obtain a sample that represents the alternative hypothesis of this study with 90% power and 95% confidence (Fleiss method with continuity correction). Considering the possibility of sample loss from the first to the second intervention, we added an additional 10% added to this sample, totaling 50 students. The sample size was calculated using OpenEpi (version 3.01) [17].

### Randomization and Allocation Concealment

The participants will be allocated randomly to one of the 2 sequences: ChatGPT (A), followed by conventional web-based tools (B), or conventional web-based tools (B), followed by ChatGPT (A). The allocation of participants into sequences (AB or BA) will be determined through computer-generated randomization in an allocation ratio of 1:1. The research team responsible for analyzing the data will be kept blind to the participants’ group assignments. The research team will generate the allocation sequence, and the TA will enroll the participants and assign them to the intervention. The trial will adhere to established procedures to maintain separation between staff that take outcome measurements and staff that deliver the intervention [13,14].

### Intervention and Measurement

During the initial phase of the study, participants assigned to sequence AB will use ChatGPT (experimental intervention: group A) to complete their assignments, while participants assigned to sequence BA will use conventional web-based tools (control intervention: group B) and serve as a control group. In the subsequent period, the interventions will switch between the 2 groups. Students will not know which one is the “intervention of interest” and which one will be the “comparator.” As mentioned above, the learning activity to be assessed in this project is part of the mandatory course activities. This means that all students will receive a grade for this assignment. Therefore, the instructor will not be able to determine which students have or have not participated in the study. Students’ acceptance to participate in this study only gives permission to the research team to use their data for research purposes, but all students will be assessed for this work as part of the course. The course instructor will not be responsible for grading the assignments associated with the intervention. The TA will be responsible for grading assignments and assigning a unique number code to each participating student, which will be linked to their names. This coded information will be sent to the RA to preserve anonymity throughout the process. The RA will receive the graded assignments, the student names, and their respective codes from the TA. The RA will collect the grades of the student participants and submit them to the research team members responsible for the analysis. It is important to note that the research team, which includes the course instructor, will not have access to the participants’ names. Only the grades and the participant codes will be accessible for analysis. The research team, including the course instructor, will perform the data analysis based solely on the provided grades and participant codes, without access to any identifiable information. This further ensures that anonymity is strictly maintained throughout the analysis phase.

### Experimental Intervention

In this study, the intervention of interest is the use of ChatGPT by students to complete their assignments. As part of this intervention, students will be given a period of 6 days to use it for assignment completion. Along with the assignment instructions, participants will receive an ethical guideline and specific guidelines on how to use ChatGPT effectively (Multimedia Appendices 1 and 2) [18-23].

### Control Intervention

In this study, the control intervention is the use of conventional web-based tools by students to complete the assignment. In this intervention, they will receive instructions on how to complete the assignment using conventional web-based tools available on the internet, without the use of ChatGPT or other generative AI platforms “and” or “or” tools. They will have a timeframe of 6 days to complete the assignment using these conventional web-based tools. Similar to the participants in the experimental intervention, they will also be required to fill out a survey on technology usability, providing feedback on their experience with the web-based tools.

### Washout Period

The purpose of the 21-day washout period is to mitigate or minimize any potential carryover effects from the intervention. The term *carryover effect* refers to the persistence of the previous intervention’s effect or the impact of the initial intervention that extends beyond its duration and influences the subsequent intervention’s effect. By incorporating a washout period, we aim to ensure a clearer distinction between the effects of each intervention and minimize any potential interference or bias [24].

The available evidence on determining the appropriate washout period to eliminate the carryover effect effectively is limited. The conventional approach for determining the washout period is often based on the concept of 5 half-lives of the specific drug involved [25]. To address the carryover effect in this study, we used 2 strategies. First, we implemented a washout period of 21 days between interventions. This time gap was intended to allow for a sufficient delay to minimize the memory of the previous intervention and any lingering effects from the previous intervention. In addition, for the second intervention, the investigators carefully selected a different topic from the assignment to ensure that students would not easily recall specific details from the previous intervention. Furthermore, the research team ensured that the assignment content for the second intervention had similar complexities and requirements to maintain consistency across the study.

### Outcomes and Measurements

The outcome measures will be (1) the usability of the technological tools (ChatGPT or conventional) for completing the assignments (primary outcome), (2) the participants’ perception of these tools as support for learning, and (3) the learning outcomes (secondary outcomes). Outcomes and measurements will be collected at 2 points: at the first follow-up, that is, after the first intervention (experimental or control) in the initial phase, and at the second follow-up, that is, after the second intervention (experimental or control) in the final phase (Figure 1).

#### Usability

Participants in the experimental intervention (AI-ChatGPT intervention) will also be asked to fill out a survey regarding their perception of using generative AI as an assistance tool to complete their assignments. This survey aims to gather insights into their thoughts, opinions, and attitudes toward using AI in their learning experiences. Participants will have the opportunity to share their feedback on the effectiveness, benefits, challenges, and overall satisfaction while using AI for assignment completion. Their responses will provide valuable information on AI technology’s perceived value and acceptance in enhancing their learning experience. Participants in the control intervention (conventional tools) will complete the same survey, but regarding the usability of the conventional tools.

The System Usability Scale (SUS) will assess the participants’ perceptions of usability. The SUS is a valid questionnaire [26] and will be administered at the end of the interventions and when participants cross over to the other assistance tool (AI-ChatGPT or conventional web-based tools without AI). The SUS is commonly used in numerous studies to assess the effectiveness and usability of new technologies, including in the education field [27-29]. A recent systematic review of home-based telerehabilitation software systems for remote supervision revealed that the SUS tool was the most frequently used measure or tool across the studies examined [28]. This scale has been translated into several languages, including the American Sign Language [30-32]. The SUS questionnaire was originally developed as a “quick and dirty” tool, with the goal of providing a fast and nonintrusive assessment experience for participants [26]. The SUS consists of 10 items formulated as affirmative statements, for which users indicate their level of agreement or disagreement on a 5-point Likert scale (1=strongly disagree; 5=strongly agree). For the odd answers (1, 3, and 5), we will subtract 1 from the score that the user answered; for the even answers (2 and 4), the score will be subtracted from 5. The values of the 10 questions will be added and multiplied by 2.5. The final score can range from 0 to 100. Internal consistency will be calculated using Cronbach α per item and overall. This scale allows for the assessment of outcomes and comparisons within and between the 2 interventions (Table 1).

**Table 1:** Overview of outcome measures.

Outcome measure		Variable or scale	Measure	Analysis method
**Primary**				
	Usability (SUS^a^)	Continuous, nominal, ordinal, and 5-point Likert-type scale^b^	0-100 (SUS score: >68=above average and <68=below average) The individual scores of the participants for each question are transformed into a new number, which is then aggregated and multiplied by 2.5 to convert the initial range of scores from 0-40 to a new range of 0-100.	The frequencies, means, and SD of each item of the SUS scale will be calculated and compared using the McNemar or chi-square and Wilcoxon tests, respectively. In addition, each group’s usability scores and grades will be correlated using Spearman correlation.
**Secondary**				
	Perception of AI^c^	Continuous, nominal, ordinal, and 5-point Likert-type scale	The questionnaire consists of four sections: (1) sociodemographic information; (2) AI literacy exploration of positive learning experiences with AI; (3) examination of negative experiences associated with AI use; and (4) an assessment of the potential for discussing accountability, fostering student leadership, and addressing ethical and equity concerns pertaining to the use of AI.	Fisher exact or Pearson chi-square test will investigate the associations between the categorized scales and sociodemographic characteristics.
Learning outcomes		Continuous	Students’ grades on both interventions. Score range (0-100)	Mean, SD

^a^SUS: System Usability Scale.

^b^5=Strongly agree, 4=agree, 3=neither agree nor disagree, 2=disagree, and 1=strongly disagree.

^c^AI: artificial intelligence.

#### Perception of Ethics and Accountability

After the intervention with ChatGPT, the students will be asked to fill out a web-based survey about their perception of their AI experience. The survey will use multiple-choice questions, short open questions and a 5-point Likert scale, using closed-ended response options. This response format ensures enhanced objectivity and enables appropriate discrimination to address the research objectives effectively. A closed-ended Likert scale also promotes the participants’ comprehension and active engagement with the study. The questionnaire consists of four sections: (1) a sociodemographic information; (2) AI literacy exploration of positive learning experiences with AI; (3) an examination of negative experiences associated with AI use; and (4) an assessment of the potential for discussing accountability, fostering student leadership, and addressing ethical and equity concerns pertaining to the use of AI. One question of this survey was taken from a Swedish questionnaire about the use and views among university students about chatbots and other AI for learning. Qualtrics software will be used for survey design and data collection, as this software passed a security assessment of Carleton University, and its server is located in Montreal, Quebec, Canada.

The SUS survey is expected to take approximately 2 minutes to complete, whereas the perception survey is anticipated to be completed in approximately 10 minutes. By collecting data at multiple points in the study (2 points), we can evaluate the effects of each intervention and any potential carryover effects during the crossover period. In addition, it provides an opportunity to measure changes in outcomes within individual participants as they switch from one intervention to another.

### Data Analysis

This study aims to compare the 2 interventions in terms of usability, student perception, and learning outcomes. The crossover design allows for within-group and between-group comparisons, enabling a comprehensive analysis of students’ perceptions of the use of the intervention and its impact on their learning, as well as the effects of the intervention on their learning outcomes.

The use of an identification code will anonymize data. The data set will be exported from the datasheet and imported into the SPSS software (version 28.0; IBM Corp) for Mac, where the data will be subjected to analysis with a confidence level of 95%. Descriptive statistics, including the calculation of frequencies, will be performed on the sociodemographic variables of the students. The usability scale and grades at each assessment time will be summarized using mean and SD. The nonparametric Wilcoxon test will be used to compare the differences between these variables.

The frequencies, means, and SDs of each item of the SUS scale will be calculated and compared using the McNemar or chi-square and Wilcoxon tests, respectively. In addition, the usability scores and the grades of each group will be correlated using Spearman correlation.

For the analysis of perception and usability outcomes, we will use the Spearman correlation coefficient to assess the relationship between the 2 scales. This statistical approach was chosen to measure the strength and direction of the associations between variables when the data may not conform to a linear pattern. Using the Spearman correlation coefficient, we aim to better understand how perceptions and usability factors are interrelated, providing a robust basis for our research findings and conclusions. To assess the internal consistency of the scales, Cronbach α coefficient will be calculated for both constructs and for each individual item.

To categorize the students into groups of low and high performance (using a cut-off value of 80) and classify the SUS scale into high and low usability groups (with a cut-off value of 50), appropriate statistical tests will be applied. Specifically, Fisher exact test or Pearson chi-square test will investigate the associations between the categorized scales and sociodemographic characteristics.

### Ethical Considerations

This study has obtained ethical approval from Carleton University Research Ethics Board-B, ethics clearance ID: 119784, ensuring strict adherence to participant confidentiality, anonymity, informed consent procedures, and data protection, in compliance with applicable institutional and ethical guidelines. No identifiable data will be included in any part of the research. Participants will be given the opportunity to enter a drawing for gift cards valued at US $25 each, specifically for completing the surveys.

## Results

Data collection took place in October and November 2023; the anticipated results are scheduled for publication in 2024. We anticipate several key findings. First, randomization is expected to have groups with closely matched demographic characteristics, ensuring a balanced starting point for our analysis. Second, we anticipate that usability will be significantly higher for the ChatGPT intervention than for conventional methods, reflecting the potential benefits of AI-assisted learning. In addition, regarding ChatGPT’s use in assignments, we expect to observe students expressing concerns about data privacy and plagiarism, consistent with prior literature findings on these issues. The potential correlation between usability and learning outcomes is a significant aspect to consider in our study. Understanding how usability impacts learning outcomes not only is critical for this study but also lays the foundation for future investigations. By quantifying the effect sizes of both the learning and usability effects in this study, we can better inform the calculation of sample sizes for larger RCTs on this topic. These data will be invaluable for designing robust experiments and ensuring that our research can provide meaningful insights into the relationship between usability and educational outcomes.

Furthermore, it is our anticipation that despite these concerns, learning outcomes will remain consistent across both interventions. We expect to observe that while these concerns may be raised, the overall effectiveness and educational benefits of ChatGPT are not compromised. Our study seeks to determine whether these anticipated results are confirmed. These data will contribute to a better understanding of the potential impacts of integrating AI into health sciences education.

This study is expected to contribute to the identification of key areas and challenges concerning the use of AI as an educational tool, specifically examining its benefits and challenges for students. By doing so, it aims to facilitate the establishment of comparable frameworks for future academic endeavors. In addition, the findings will enlighten educators about the potential advantages and barriers associated with integrating AI into educational activities for university students.

We expect this study’s findings to be disseminated through multiple channels. The study will undergo a rigorous peer review process for publication in a reputable, peer-reviewed journal. Subsequently, the results will be initially shared with the community in the health sciences department, ensuring timely access for the scientific community. Furthermore, we intend to submit the results of this study to relevant national conferences to provide a platform for knowledge exchange and engagement with a broader audience.

## Discussion

### Overview

This study will compare the use of ChatGPT and web-based conventional tools in terms of usability, student perception, and learning outcomes. ChatGPT is expected to be perceived as more usable than conventional tools. However, we anticipate that both groups will achieve similar learning outcomes. Furthermore, our findings may align with those of previous studies that underscore the significance of AI in education, albeit with concerns related to data privacy, as students increasingly recognize the importance of AI in their learning experiences.

This study is expected to provide details on students’ perceptions of using AI to perform their learning assignments. The results of this study will be compared with those of the literature. In recent years, there has been a growing body of research on AI, focusing on its applications in various fields, including medical education, health care, radiography, and ophthalmology [5,33,34]. Many of these studies use cross-sectional and qualitative research methods to explore the perspectives and attitudes of professionals and students [33,35,36]. Despite variations in the specific field of study, some common themes emerge from this research. These themes include a generally positive attitude toward AI’s potential benefits, a widespread lack of formal AI education among participants, concerns about data privacy and ethical implications, and a clear and urgent need for structured AI education to be integrated into relevant curricula [33,35]. These studies collectively underscore the importance of preparing individuals in these fields for the increasing role of AI in their respective domains.

A study focused on assessing medical students’ knowledge, attitudes, and perceptions of AI chatbots in health care revealed that while medical students generally held positive attitudes toward AI, they also had data protection and monitoring concerns. Students expressed a desire for more comprehensive AI education integrated into their medical curriculum, emphasizing the importance of preparing them for AI’s role in health care [35]. A systematic review of the utility of ChatGPT, an AI-based conversational large language model, in health care education, research, and practice highlighted numerous benefits and emphasized the need for a code of ethics and responsible use guidelines for AI chatbots, such as ChatGPT in health care and academia, to maximize their potential while addressing limitations and risks [8]. A Canadian study explored how undergraduate medical students in Canada perceive AI in medical education. The survey conducted with 486 students from all 17 Canadian medical schools revealed that while there was a consensus that AI will be important in future medicine and should be formally taught, many students believed that there are currently insufficient educational opportunities related to AI. Interviews with students echoed these concerns, emphasizing the need for AI integration into the medical curriculum to better prepare future physicians for the changing health care landscape [33].

These studies have provided valuable insights into students’ and professionals’ knowledge, attitudes, and perceptions regarding AI and AI chatbots in health care and related fields. They generally indicate positive attitudes toward AI’s potential benefits, along with concerns about data privacy and the need for structured AI education. However, it is essential to acknowledge that these studies have limitations, including potential biases inherent to their study designs. Therefore, further research is needed to delve deeper into the utility and students’ perceptions of AI chatbots, such as ChatGPT, in health care and academia. To address these gaps and gain a more comprehensive understanding, we plan to conduct a crossover RCT. This proposed study will enable us to systematically investigate the effectiveness and user perceptions of ChatGPT in specific health sciences and educational contexts while controlling for potential biases and limitations observed in previous studies.

### Limitations and Strengths

Although this study possesses several strengths, it anticipates limitations. First, the sample size may restrict the generalizability of the findings to a broader population of health sciences students. However, this constraint is balanced by the advantages of a focused investigation within a specific academic institution, allowing for in-depth exploration. Second, the relatively short intervention period may limit the ability to capture long-term effects or changes in participants’ perceptions over time. Nevertheless, this controlled timeframe allows for a rigorous assessment of immediate impacts. Moreover, although the study specifically examines the use of ChatGPT, acknowledging the limitation of generalizability to other AI tools, it contributes to the existing body of knowledge in this field. Finally, participants’ self-reported data, including their perceptions and experiences, may introduce response bias. However, this method aligns with the quantitative nature of the research and provides rich insights into participants’ perspectives. Despite these anticipated limitations, this study’s strengths lie in its focused investigation, controlled timeframe, and quantitative approach, all of which contribute valuable insights into the usability, perceptions, and potential benefits of using AI in health sciences education.

It is important to note that the next phase of this study, which involves subsequent qualitative data collection and analysis, is strategically designed to address some of these limitations. By investigating participants’ experiences and perceptions through focus groups, the next phase of the study aims to provide a more comprehensive understanding of the long-term effects, nuanced challenges, and potential benefits associated with AI use in health sciences education. This approach complements the quantitative phase of the study presented in this protocol and enhances the overall robustness of the study’s findings, thereby mitigating some of the anticipated limitations.

## Appendix

Multimedia Appendix 1 Tips to interact with artificial intelligence.

Multimedia Appendix 2 Guidelines for equitable use of artificial intelligence.

## Abbreviations

AI: artificial intelligence
CONSORT: Consolidated Standards of Reporting Trials
RA: research assistant
RCT: randomized controlled trial
SUS: System Usability Scale
TA: teaching assistant

## Data Availability

The data sets used and analyzed during this study are available from the corresponding author upon reasonable request.
