# Supplementary material for: Usability and Efficacy of Artificial Intelligence Chatbots (ChatGPT) for Health Sciences Students: Protocol for a Crossover Randomized Controlled Trial
Source: JMIR Res Protoc. 2023 Nov 24;12:e51873. doi: 10.2196/51873 (PMC10709780; doi:10.2196/51873)
Supplement: Multimedia Appendix 1 [file resprot_v12i1e51873_app1.docx]

## **TIPS TO INTERACT WITH ARTIFICIAL INTELLIGENCE (AI) CHATGPT**

**TIPS TO INTERACT WITH ARTIFICIAL INTELLIGENCE (AI) CHATGPT**

**Author:** Mirella Veras, PT, Ph.D.

1. **Define your objective:** clarify what you want from your AI (brainstorm, the structure of a project, specific search, etc.). You can start with a clear and relevant prompt to your assignment and then ask follow-up questions.
2. **Use your leadership skills.** The use of AI can help you to build your leadership skills. Talk to AI as if you were asking a personal assistant to help you to complete a task by using the 3 D’s (**D**iving in, **D**elegating and **D**irecting).
   1. **Diving in (Engaging)** refers to actively interacting with artificial intelligence systems. It involves immersing oneself in the process, exploring the capabilities of AI, and understanding its potential applications. Engaging with AI includes learning about its features, limitations, and ethical considerations, enabling users to make informed decisions and harness its power effectively.
   2. **Delegating:** Delegating remains relatively unchanged in the context of AI. It refers to assigning tasks or responsibilities to artificial intelligence systems, leveraging their computational abilities to perform complex operations more efficiently and accurately. Delegating to AI involves identifying suitable functions that align with its strengths, setting clear objectives, and ensuring appropriate monitoring and evaluation to maintain desired outcomes.
   3. **Directing:** In artificial Intelligence, Directing: In artificial intelligence, "Directing" can be redefined as "Guiding." This involves providing AI systems with instructions, guidelines, and constraints to achieve specific goals. Provide context by giving more details on your prompts. If you are unsatisfied with the response, give more information and ask follow-up questions. Ensure you don’t share sensitive, personal or private information. Guiding AI requires a deep understanding of the problem domain and the technology's capabilities. It entails formulating strategies, defining objectives, and continuously monitoring and adjusting the AI systems' behaviour to align with desired outcomes and ethical standards. Adopting these redefined 3 D's (Diving in, Delegating, and Directing) allows you to effectively interact with artificial intelligence, harness its capabilities, and ensure responsible and beneficial integration into various domains.
3. Use step by step on your prompt instructions and provide a “to-do list” to your assistant.
4. Limit your response length to the number of words you need.
5. Rephrase your questions, as needed, until you are satisfied with the outcomes.
