# Supplementary material for: Usability and Efficacy of Artificial Intelligence Chatbots (ChatGPT) for Health Sciences Students: Protocol for a Crossover Randomized Controlled Trial
Source: JMIR Res Protoc. 2023 Nov 24;12:e51873. doi: 10.2196/51873 (PMC10709780; doi:10.2196/51873)
Supplement: Multimedia Appendix 2 [file resprot_v12i1e51873_app2.pdf]

APPENDIX A: GUIDELINE FOR ETHICAL AND EQUITABLE USE OF AI

GUIDELINES FOR ETHICAL AND EQUITABLE USE OF AI

Author: Mirella Veras, PT, Ph.D.

Students in this course can use AI responsibly and ethically, but they need to follow specific guidelines for each assignment. This Fall term 2023, the only assignment that allows the use of AI is assignment #1, and students need to follow the specific guidelines for assignment #1. Understanding the potential benefits and the advances of the technology, but also the potential misuse of the tool, I have outlined here a general ethical and responsible guideline for the use of AI:

1. **AI Digital Literacy:** Students should understand AI technologies to make informed decisions about their use. The knowledge includes competence in engaging with AI, including how to effectively communicate with AI, and recognize its limitations, bias, risks, capabilities, and potential positive and negative impacts on student learning and society.

2. **Responsible use:** Students should use their critical thinking abilities to understand the value of the information provided by the AI and relate it to their context, verify and evaluate the information from AI prompts and compare it with other sources. Some examples of the responsible use of AI are editing, translating, outlining, brainstorming for the first draft and revising. The use of AI outside these examples is considered academic dishonesty.

3. **Citation:** No citation is required if a student uses AI to improve their learning and optimize their time spent searching in general web browsing without directly using the AI's information. Example: Asking AI for examples of the most reputable databases for psychology research or asking AI where to find official information about statistics on diabetes in Canada. Outside of these examples, students should support academic integrity by appropriately citing sources and completing their assignments. The use of AI should be mentioned in the references.
   If a student uses AI, you should reference it as per these examples.
   As per information from Dalhousie University:

“Currently, APA recommends that text generated from AI be formatted as "Personal Communication." As such, it receives an in-text citation but not an entry on the References list.”

Rule: (Communicator, personal communication, Month Date, Year)

Examples:

(OpenAI, personal communication, January 16, 2023).

When asked to explain psychology's main schools of thought, OpenAI's ChatGPT's response included ... (personal communication, February 22, 2023).

Vancouver style

“The NLM Style Guide recommends that personal communications *are only cited in the text, and are not included in the reference list. Generally, the Vancouver referencing style uses numbered in-text citations, but for personal communications the advice is as follows:*

provide information about the personal communication within the text
Include the nature and source of the cited information
indicate clearly that no corresponding citation is in the reference list (e.g. include the term 'unreferenced' or similar)

Example: In response to the question ..., OpenAI's ChatGPT gave the following response ... (2023 Feb 22, unreferenced).”

4. **Security and Privacy:** Students should not use personal information of themselves or others and prioritize the protection of personal data. It is important to know how the AI collects, secures and stores the information you input on the platform.

5. **Bias and Discrimination:** Students should critically evaluate AI systems' outcomes and recommendations and identify potential risks for bias and discrimination.

6. **Beneficence:** Students should leverage the potential of AI to enhance their learning journey while striving to make meaningful contributions that positively impact society as a whole.

7. **Non-maleficence:** Students should employ AI in an ethical manner, refraining from using it for malicious purposes or spreading misinformation. It is essential to prioritize the responsible and accountable use of AI.

8. **Autonomy:** Students should use AI to enhance their autonomy and maintain control over their learning activities to avoid excessive dependence on the system. By responsibly leveraging AI tools and technologies, students can empower themselves to take ownership of their learning process while utilizing AI as a supportive resource. Striving for a balanced approach, students can benefit from the capabilities of AI without surrendering their independent thinking and decision-making skills.

By adhering to these guidelines, students can promote the ethical use of AI, contribute to responsible and ethical AI use and ensure that AI technologies are utilized in a manner that benefits both individuals and society as a whole. As part of this exercise, I asked OpenAI's ChatGPT's about their thoughts on students using AI for their assignments, and the answer to my question was:

"As an AI language model, I don't have personal opinions or thoughts. However, I can provide information on the topic. The use of AI in assignments can offer various advantages for students. It can facilitate research, data analysis, and organization of information, potentially saving time and enhancing the quality of work. AI tools can assist in proofreading, grammar checking, and generating ideas. However, it is essential for students to ensure that they use AI tools ethically, following academic integrity guidelines and acknowledging the contribution of AI in their work. Ultimately, the decision to use AI in assignments should be based on the specific requirements, guidelines, and ethical considerations set by educational institutions." I must say that AI pointed out the important benefits of its responsible use. Please use this guideline to address any potential positive or negative impact of the use of AI in your learning journey in this course.

References:

A.B. Brendel, M. Mirbabaie, T.B.Lembcke, L. Hofeditz. Ethical management of artificial intelligence Sustainability, 13 (4) (2021), pp. 1-18.

Yi, Yumi. Establishing the concept of AI literacy: Focusing on competence and purpose. JAHR, Vol. 12/2, No. 24 2021 Preliminary communication / Prethodno priopćenje, Received / Zaprimljen: 19. 3. 2021.

McMaster University, Appendix B. Sample McMaster Syllabus statement. Available at: https://mi.mcmaster.ca/generative-artificial-intelligence-in-teaching-and-learning/#tab-content-provisional-guidelines. Accessed on June 17, 2023.

Dalhousie University. Citation Style Guide. Available at:https://dal.ca.libguides.com/CitationStyleGuide/citing-ai#:~:text=Currently%2C%20APA%20recommends%20that%20text,entry%20on%20the%20References%20list.&text=Examples%3A,%2C%20January%2016%2C%202023). Accessed on June 17, 2023.

Kong, S.-C., Cheung, W. M.-Y., & Zhang, G. (2023). Evaluating an Artificial Intelligence Literacy Programme for Developing University Students' Conceptual Understanding, Literacy, Empowerment and Ethical Awareness. *Educational Technology & Society*, *26*(1), 16-30. https://doi.org/10.30191/ETS.202301_26(1).0002

OpenAI, personal communication, June 17, 2023
